# Association of plasma trans fatty acid concentrations with blood pressure and hypertension in U.S. adults

**DOI:** 10.3389/fendo.2024.1373095

**Published:** 2024-04-22

**Authors:** Min Luan, Youping Tian, Dandan Yan, Shuang Liang

**Affiliations:** ^1^ Clinical Research Center, Shanghai Sixth People’s Hospital Affiliated to Shanghai Jiao Tong University School of Medicine, Shanghai, China; ^2^ National Management Office of Neonatal Screening Project for Congenital Heart Disease (CHD), Children’s Hospital of Fudan University, National Children’s Medical Center, Shanghai, China; ^3^ Department of Endocrinology and Metabolism, Shanghai Sixth People’s Hospital Affiliated to Shanghai Jiao Tong University School of Medicine, Shanghai Diabetes Institute, Shanghai Clinical Center of Diabetes, Shanghai Key Laboratory of Diabetes Mellitus, Shanghai Key Clinical Center for Metabolic Disease, Shanghai, China; ^4^ Department of Obstetrics and Gynecology, Shanghai Sixth People’s Hospital Affiliated to Shanghai Jiao Tong University School of Medicine, Shanghai, China

**Keywords:** hypertension, NHANES, trans fatty acids, Bayesian Kernel Machine Regression, blood pressure, United States

## Abstract

**Objective:**

The present study aimed to evaluate the association of plasma trans fatty acids (TFAs) biomarkers with the risk of hypertension.

**Methods:**

Using data from the National Health and Nutrition Examination Surveys (NHANES 2009-2010), we conducted a thorough analysis using both the traditional regression model and the Bayesian Kernel Machine Regression (BKMR) model to investigate the associations of individual TFAs and their mixtures with systolic blood pressure (SBP), diastolic blood pressure (DBP), and the risk of hypertension in a sample of 1,970 American adults.

**Results:**

The concentrations of TFAs were natural logarithms (ln) transformed to approximate a normal distribution. Multivariate linear regression models showed that each 1-unit increase in ln-transformed plasma concentrations of palmitelaidic, elaidic, vaccenic, and linolelaidic acids was associated with separate 2.94-, 3.60-, 2.46- and 4.78-mm Hg and 2.77-, 2.35-, 2.03-, and 3.70- mm Hg increase in SBP and DBP, respectively (*P* < 0.05). The BKMR model showed positive associations between the four TFAs mixtures and SBP and DBP. In addition, linolelaidic acid contributed the most to an increased blood pressure. Similar results were observed with the threshold of hypertension (≥130/80 mm Hg).

**Conclusion:**

Our findings provide preliminary evidence that plasma TFA concentrations are associated with increased blood pressure and the risk of hypertension in US adults. This study also suggests that linolelaidic acid might exhibit more deleterious effects on hypertension than other TFAs. Further studies should be conducted to validate these results.

## 1 Introduction

Hypertension poses a major global public health challenge (1). Its prevalence has steadily increased worldwide in recent decades, and serves as a major risk factor for chronic kidney disease (CKD), cardiovascular diseases (CVD), and mortality (2–4). In 2018, approximately half of all adults in the US were affected by hypertension (5). Furthermore, the total number of hypertension-related CVD deaths increased from 171,259 in 2000 to 270,839 in 2018 (6). Accumulating evidence has revealed that genetic predisposition, diet, physical activity, smoking, and alcohol consumption are major risk factors for hypertension (7). Among these, diet is a modifiable and preventable factor for disease risk and has garnered increasing attention.

Trans fatty acids (TFAs) are unsaturated fatty acids characterized by one or more carbon-carbon double bonds in the trans configuration (8) and are commonly used as food additives in margarines, snack foods, packaged baked goods, and fried foods (9). Several types of TFAs are produced during the partial hydrogenation of vegetable and seed oils, including the trans-isomers of oleic acid (trans-18:1) and linoleic acid (trans-18:2). Ruminant bacteria also produce small amounts of palmitoleic acid trans-isomers (trans-16:1) (10). Since humans do not synthesize TFAs, exposure to these compounds typically occurs through the consumption of industrially processed high-fat foods (11). *In vivo* and *in vitro* studies suggest that industrial TFAs can induce inflammation and oxidative stress (12), whereas inflammasome activation and endoplasmic reticular stress play important roles in the progression of hypertension (13, 14). However, population-based studies on the association between plasma TFAs biomarkers and the risk of hypertension are insufficient and inconsistent. Several studies have reported the adverse effects of TFAs on blood pressure, whereas other studies have reported protective associations between TFAs and blood pressure (15, 16). Humans are exposed to numerous TFAs in their daily lives. TFA subtypes and their specific isomers may have different adverse health effects (17). The available evidence on the effects of mixed TFAs exposure and the single effects of individual TFA on blood pressure remains limited, and further studies are urgently warranted.

Using data from the National Health and Nutrition Examination Surveys (NHANES) 2009-2010, we used a traditional regression model to evaluate the associations of TFAs concentrations with blood pressure and the risk of hypertension, and further performed Bayesian Kernel Machine Regression (BKMR) model to assess the overall associations of TFAs with blood pressure and the risk of hypertension in the US adults.

## 2 Materials and methods

### 2.1 Study population

The NHANES is a series of nationwide cross-sectional surveys on the health and nutrition of the US population conducted by the National Center for Health Statistics (NCHS) in a 2-year cycle. This study was approved by the NCHS Research Ethics Review Board, and all participants provided written informed consent. More detailed information on the NHANES can be found on the official website: www.cdc.gov/nchs/nhanes/.

The current study used data from the 2009 to 2010 NHANES cycles. A total of 5,793 participants aged 20-79 years were recruited. Of these participants, 2,325 had available data on plasma TFAs biomarkers and blood pressure measurements. Participants who were pregnant (n = 26) or had incomplete covariate data (n = 329) were excluded. A total of 1,970 participants were included in this study (**Supplementary Figure S1**).

### 2.2 Plasma TFAs measurements

The four TFAs were analyzed in plasma using isotope dilution-gas chromatography-negative chemical ionization-mass spectrometry (ID-GC-NCI-MS), as detailed in a previous study (18). These TFAs include *trans*-9-hexadecenoic acid (palmitelaidic acid, C16:1n-7t), *trans*-9-octadecenoic acid (elaidic acid, C18:1n-9t), *trans*-11-octadecenoic acid (vaccenic acid, C18:1n-7t), and *trans*-9, *trans*-12-octadecadienoic acid (linolelaidic acid, C18:2n-6t, 9t), and are the most prevalent TFAs in foods, accounting for about 40–60% of total TFAs present in the human body (19).

### 2.3 Outcomes

Blood pressure measurements were performed by trained examiners using a mercury sphygmomanometer, following established protocols. After 5 min of rest in a seated position, the participant’s maximum inflation level was established, and blood pressure was subsequently measured on the same arm three times consecutively. Average systolic and diastolic blood pressures (SBP and DBP, respectively) were calculated.

Based on the 2017 American College of Cardiology (ACC)/American Heart Association (AHA) blood pressure guideline (7), hypertension was defined as the average SBP being ≥130 mm Hg, or the average DBP ≥80 mm Hg. If a participant answered “yes” to the question ‘Are you now taking prescribed medicine for high blood pressure?’ or self-reported hypertension was considered hypertensive.

### 2.4 Covariates

The potential confounders for this study were selected *a priori* from previous studies on TFAs and hypertension (20). This study collected data on demographic variables such as age, sex, race/ethnicity, education level, and poverty income ratio (PIR) through household interviews and questionnaires. Educational level was categorized as follows: less than high school, high school graduate or equivalent, and college or above. The PIR, an index of the ratio of family income to poverty, was calculated by dividing family income by poverty guidelines specific to family size (21). The participants’ weights and heights were obtained from examination data. Body mass index (BMI) was calculated as weight in kilograms divided by height in meters squared (< 25 kg/m^2^, 25–30 kg/m^2^, and ≥30 kg/m^2^). Data on smoking status (smoking at least 100 cigarettes in life or not), alcohol consumption (at least 12 alcohol drinks/1 year or not), and recreational physical activity (vigorous, moderate, or no activity) were acquired from the questionnaire data. Diabetes was defined as meeting one of the following four conditions: a) glycated hemoglobin > 6.5%; b) fasting blood glucose >126 mg/dL; c) 2 h plasma glucose ≥200 mg/dL, and d) a self-report of a diagnosis by a physician or health care professional (22). The estimated glomerular filtration rate (eGFR) was calculated using new creatinine- and cystatin C–based equations (23). We defined CKD as either individuals developing an eGFR ≤60 mL/min/1.73 m^2^ or urine albumin-creatinine ratio ≥30 mg/g. A history of atherosclerotic cardiovascular disease (ASCVD) was defined as at least one diagnosis of coronary heart disease, angina, heart attack, or stroke.

### 2.5 Statistical analyses

Participants’ demographic characteristics were presented with the mean ± standard deviation (SD) for continuous variables, and counts (percentages) for categorical variables. We used the geometric mean (GM), geometric standard deviations (GSD), and percentiles to characterize the distributions of plasma TFA concentrations. TFA concentrations were natural log (ln)-transformed to approximate a normal distribution. Pearson’s correlation was used to assess the correlation between pairs of ln-transformed TFA concentrations.

We initially used the restricted cubic spline (RCS) model with three knots at the 10^th^, 50^th^, and 90^th^ percentiles to evaluate the non-linear associations between plasma TFA concentrations and SBP and DBP. We used multivariate linear regression models to examine the association between ln-transformed TFA concentrations (continuous variables) and SBP and DBP. Given that a few associations were non-linear (*P*-value in the RCS < 0.10, as shown in **Supplementary Figure S2**), we categorized plasma TFA concentrations by quartiles to evaluate the nonmonotonic associations of plasma TFA concentrations with SBP and DBP in multivariate linear regression models. Hypertension has a high prevalence in U.S. adults (approximately 50%) (5); therefore, we used a modified Poisson regression model with robust variance estimates to evaluate the association between TFA exposure and the risk of hypertension (24).

We used the BKMR model to evaluate the single and overall effects of exposure to the four TFAs on blood pressure and hypertension. This approach combines Bayesian and statistical learning methods to iteratively regress the response variable on the non-parametric terms of multiple exposure mixture components, allowing for potential non-linear and non-additive effects (25). The base BKMR model was used to evaluate the associations of the four TFAs with the continuous outcomes of blood pressure measurements, whereas the BKMR-P model with a probit link function was used to examine the effect of the four TFAs on hypertension defined by clinical threshold values. All BKMR models were constructed using a Markov Chain Monte Carlo (MCMC) with 10,000 iterations. The posterior inclusion probabilities (PIPs) were calculated to indicate the highest TFA within the mixture. A PIP value of ≥ 0.5 was considered the threshold value (26).

The overall associations of the TFAs mixture with SBP and DBP and the risk of hypertension were visualized by depicting the differences (95% credible intervals, CrI) in SBP, DBP and hypertension, holding the plasma concentrations of all TFAs at the same percentiles (30^th^, 35^th^, 40^th^, 45^th^, 50^th^, 55^th^, 60^th^, 65^th^, 70^th^, and 75^th^ percentiles) compared to those at the 25^th^ percentile. The single effects of TFAs on SBP, DBP, and hypertension were presented as the estimated changes (95% CrI) in these outcomes for the changes in each TFA concentration between the 25^th^ and 75^th^, while simultaneously fixing the other TFAs at the 25^th^, 50^th^, and 75^th^ percentiles.

We performed several sensitivity analyses. Considering the complexity of the sampling design, we applied survey-weighted linear regression analyses to evaluate the associations of TFAs with SBP and DBP to create nationally representative estimates. Secondly, it is reported that plasma TFA concentrations decreased by 54% from 1999-2000 to 2009-2010 (16). We repeated the main analyses among the population in 1999-2000, which facilitated to understand some patterns of the effects of TFA exposure on blood pressure in the light of different levels. We additionally adjusted the associations for ASCVD history to test the robustness of the results.

SAS 9.4 (SAS Institute Inc., Cary, NC, USA) and R 4.2.2 (R Development Core Team) were used for statistical analyses. The BKMR was implemented with the R packages “bkmr” and “ggplot2”. A *P*-value of < 0.05 is considered statistically significant.

## 3 Results

### 3.1 Study population characteristics

The demographic characteristics of the 1,970 US adults are presented in **Table 1**. In the total population, the prevalence of hypertension was 48.68%, with an average age of 47.94 ± 16.37 years. Approximately half of the participants were males (50.02%), non-Hispanic white (49.34%), had a PIR greater than 1.85 (48.58%), and reported no recreational activities (51.52%). Most adults were overweight or obese (73.05%) and had some college education or higher level of education (73.71%). Among the participants, 45.84% were smokers, 74.47% were alcohol drinkers, and ≥ 10% had diabetes (11.32%) and CKD (12.49%). Except for PIR, significant differences were noted between the hypertensive and non-hypertensive adults in terms of age, sex, race/ethnicity, BMI, education level, recreational activities, smoking, drinking, diabetes, and CKD (all *P*-value < 0.05).

**Table 1:** Characteristics of the study population.

Characteristics	Study population (N=1970)	Hypertension		*P*-value
		No (N=1011)	Yes (N=959)	
Age (mean ± SD, years)	47.94 ± 16.37	40.60 ± 14.61	55.69 ± 14.45	<0.01
Sex (n, %)				
Male	1485 (50.02)	1133 (48.61)	352 (55.17)	<0.01
Female	1484 (49.98)	1198 (51.39)	286 (44.83)	
Race (n, %)				
Mexican American	373 (18.93)	223 (22.06)	150 (15.64)	<0.01
Other Hispanic	201 (10.20)	115 (11.37)	86 (8.97)	
Non-Hispanic White	972 (49.34)	509 (50.35)	463 (48.28)	
Non-Hispanic Black	336 (17.06)	120 (11.87)	216 (22.52)	
Other Races	88 (4.47)	44 (4.35)	44 (4.59)	
Body mass index categories (n, %)				
Normal (<25 kg/m^2^)	531 (26.95)	368 (36.40)	163 (17.00)	<0.01
Overweight (25–30 kg/m^2^)	664 (33.71)	367 (36.30)	297 (30.97)	
Obesity (≥30 kg/m^2^)	775 (39.34)	276 (27.30)	499 (52.03)	
Educational level (n, %)				
Less than high school	208 (10.56)	108 (10.68)	100 (10.43)	<0.01
High school graduate/GED or equivalent	310 (15.74)	134 (13.25)	176 (18.35)	
College or above	1452 (73.71)	769 (76.06)	683 (71.22)	
Poverty income ratio (n, %)				
≤1.30	712 (36.14)	369 (36.5)	343 (35.77)	0.51
1.30–1.85	301 (15.28)	162 (16.02)	139 (14.49)	
≥1.85	957 (48.58)	480 (47.48)	477 (49.74)	
Recreational activities (n, %)				
No	1015 (51.52)	458 (45.30)	557 (58.08)	<0.01
Moderate	404 (20.51)	281 (27.79)	123 (12.83)	
Vigorous	551 (27.97)	272 (26.90)	279 (29.09)	
Smoking status (n, %)				
No	1067 (54.16)	571 (56.48)	496 (51.72)	0.03
Yes	903 (45.84)	440 (43.52)	463 (48.28)	
Drinking status (n, %)				
No	503 (25.53)	211 (20.87)	292 (30.45)	<0.01
Yes	1467 (74.47)	800 (79.13)	667 (69.55)	
Diabetes (n, %)				
No	1747 (88.68)	964 (95.35)	783 (81.65)	<0.01
Yes	223 (11.32)	47 (4.65)	176 (18.35)	
Chronic kidney disease (n, %)				
No	1724 (87.51)	950 (93.97)	774 (80.71)	<0.01
Yes	246 (12.49)	61 (6.03)	185 (19.29)	
History of atherosclerosis cardiovascular disease				
No	1801 (91.75)	981 (97.42)	820 (85.77)	<0.01
Yes	162 (8.25)	26 (2.58)	136 (14.23)	

### 3.2 Distributions of plasma TFA concentrations

**Table 2** presents the distributions of the plasma TFA concentrations. All four TFAs were detected in plasma samples of US adults. Vaccenic acid had the highest median (interquartile range) concentration (18.10 [13.30–24.90] µmol/L), followed by elaidic, palmitelaidic, and linolelaidic acids. Plasma concentrations of four TFAs were highly correlated with each other (r = 0.60–0.86, *P*-value < 0.001, **Supplementary Figure S3**).

**Table 2:** Distributions of plasma trans fatty acid concentrations.

Trans fatty acid (umol/L)	GM (GSD)	5^th^	10^th^	25^th^	50^th^	75^th^	90^th^	95^th^
Palmitelaidic acid	3.85 ± 1.54	1.97	2.24	2.88	3.83	5.12	6.71	7.72
Vaccenic acid	18.30 ± 1.66	8.05	9.78	13.30	18.10	24.90	34.10	42.80
Elaidic acid	13.88 ± 1.70	6.07	6.99	9.56	13.50	19.30	27.70	35.00
Linolelaidic acid	1.61 ± 1.56	0.82	0.93	1.18	1.58	2.15	2.84	3.41

GM, geometric mean; GSD, geometric standard deviation.

### 3.3 Associations of TFAs with blood pressure in multivariate linear regression models

ln-transformed TFA concentrations were associated with increased SBP and DBP (**Table 3**). Specifically, for per unit increase in ln-transformed plasma concentrations of palmitelaidic, elaidic, vaccenic, and linolelaidic acids, SBP increased by 2.94 (95% confidence interval (CI): 1.27, 4.60), 3.60 (95% CI: 2.21, 4.99), 2.46 (95% CI: 1.07, 3.84), and 4.78 (95% CI: 3.10, 6.46) mm Hg, respectively, and DBP increased by 2.77 (95% CI: 1.53, 4.01), 2.35 (95% CI: 1.32, 3.38), 2.03 (95% CI: 1.00, 3.06), and 3.70 (95% CI: 2.45, 4.95) mm Hg, respectively. Estimates for TFAs, modeled as quartiles, showed similar patterns of positive associations. Higher exposures to palmitelaidic, elaidic, vaccenic, and linolelaidic acids were associated with increased SBP and DBP. Moreover, the highest quartiles carried the highest risk with estimates ranging 2.39–6.01 (**Table 3**).

**Table 3:** Associations of plasma trans fatty acid concentrations with systolic blood pressure and diastolic blood pressure in multivariate linear regression models.

Trans fatty acid	Systolic blood pressure		Diastolic blood pressure	
	β (95% CI)*	P-value	β (95% CI)*	P-value
Palmitelaidic acid				
Quartile1	Ref	Ref	Ref	Ref
Quartile2	-1.09 (-3.01, 0.83)	0.27	-0.08 (-1.51, 1.34)	0.91
Quartile3	0.49 (-1.47, 2.45)	0.62	1.56 (0.10, 3.02)	0.04
Quartile4	2.39 (0.40, 4.39)	0.02	2.66 (1.18, 4.15)	<0.01
Continuous	2.94 (1.27, 4.60)	<0.01	2.77 (1.53, 4.01)	<0.01
Vaccenic acid				
Quartile1	Ref	Ref	Ref	Ref
Quartile2	-0.16 (-2.08, 1.76)	0.87	1.24 (-0.19, 2.67)	0.09
Quartile3	0.59 (-1.35, 2.53)	0.55	2.27 (0.83, 3.72)	<0.01
Quartile4	3.07 (1.09, 5.05)	<0.01	2.64 (1.17, 4.12)	<0.01
Continuous	2.46 (1.07, 3.84)	<0.01	2.03 (1.00, 3.06)	<0.01
Elaidic acid				
Quartile1	Ref	Ref	Ref	Ref
Quartile2	2.45 (0.51, 4.39)	0.01	1.08 (-0.36, 2.53)	0.14
Quartile3	1.08 (-0.91, 3.07)	0.29	2.00 (0.53, 3.47)	<0.01
Quartile4	4.83 (2.76, 6.90)	<0.01	3.49 (1.95, 5.03)	<0.01
Continuous	3.60 (2.21, 4.99)	<0.01	2.35 (1.32, 3.38)	<0.01
Linolelaidic acid				
Quartile1	Ref	Ref	Ref	Ref
Quartile2	2.54 (0.56, 4.51)	0.01	2.51 (1.04, 3.98)	<0.01
Quartile3	2.16 (0.14, 4.18)	0.04	2.05 (0.54, 3.55)	<0.01
Quartile4	6.01 (3.93, 8.09)	<0.01	4.45 (2.90, 5.99)	<0.01
Continuous	4.78 (3.10, 6.46)	<0.01	3.70 (2.45, 4.95)	<0.01

*Adjusting for age, sex, race/ethnicity, education levels, poverty income ratio, body mass index, smoking status), alcohol consumption, recreational physical activity, history of diabetes, and chronic kidney disease history.

### 3.4 Associations of TFAs with blood pressure in the BKMR model

**Figure 1:**
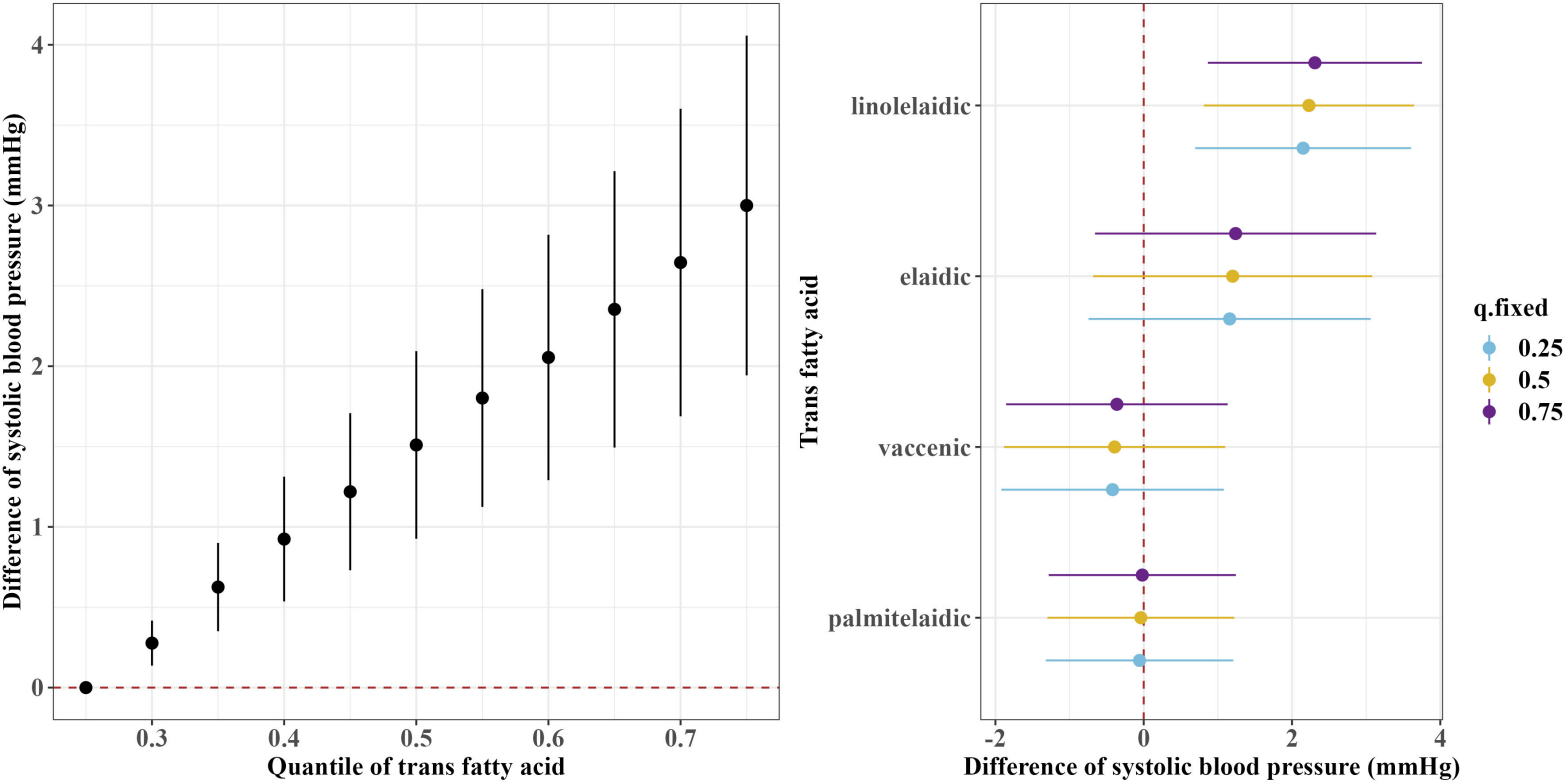
Overall and single-exposure effects of trans fatty acid on systolic blood pressure in Bayesian kernel machine regression models. The left figure shows the effects of four trans fatty acids mixture exposure on systolic blood pressure. The overall associations of TFAs mixture with systolic blood pressure were visualized by the point estimates and their 95% CrI for the difference in systolic blood pressure when all TFAs in the mixture were fixed at the 25^th^ and 75^th^ percentile (per 5 percentile interval), as compared to when they were all fixed at their 25^th^ percentile. The right figure shows the single-exposure effect of TFAs on systolic blood pressure. The single-exposure effect show the specific point estimates and their 95% CrI for the difference in systolic blood pressure for an interquartile range in individual TFA between the 25^th^ and 75^th^ percentile when all the other trans fatty acid were fixed at either the 25^th^ (blue line), 50^th^ (yellow line), or 75^th^ percentile (purple line). All models included the random intercept and adjusted for age, sex, race/ethnicity, education levels, poverty income ratio, body mass index, smoking status), alcohol consumption, recreational physical activity, history of diabetes, and chronic kidney disease history.

**Figure 2:**
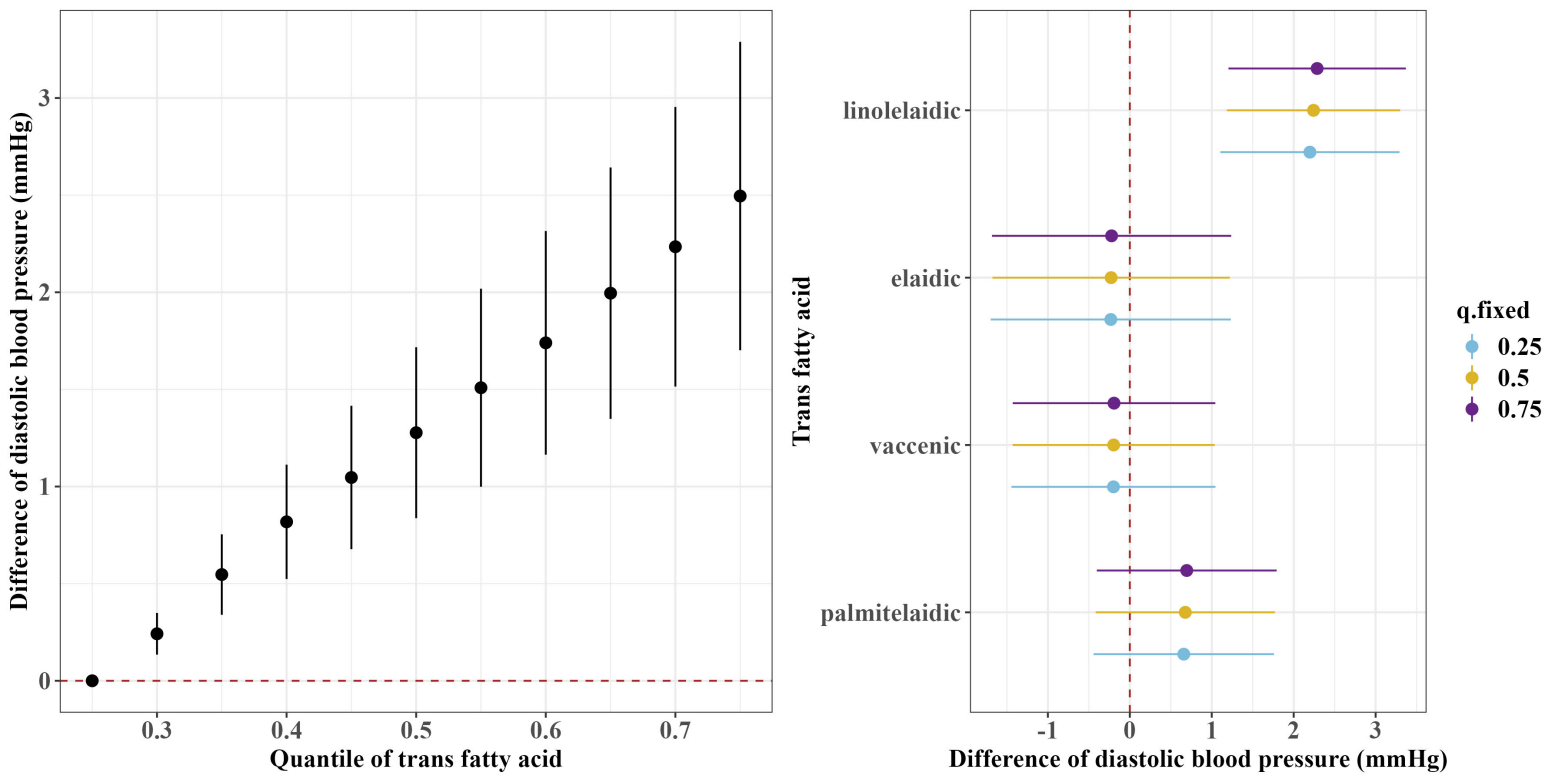
Overall and single-exposure effects of trans fatty acid on diastolic blood pressure in Bayesian kernel machine regression models. The left figure shows the effects of four trans fatty acids mixture exposure on diastolic blood pressure. The overall associations of TFAs mixture with diastolic blood pressure were visualized by the point estimates and their 95% CrI for the difference in diastolic blood pressure when all TFAs in the mixture were fixed at the 25^th^ and 75^th^ percentile (per 5 percentile interval), as compared to when they were all fixed at their 25^th^ percentile. The right figure shows the single-exposure effect of TFAs on diastolic blood pressure. The single-exposure effect show the specific point estimates and their 95% CrI for the difference in diastolic blood pressure for an interquartile range in individual TFA between the 25^th^ and 75^th^ percentile when all the other trans fatty acid were fixed at either the 25^th^ (blue line), 50^th^ (yellow line), or 75^th^ percentile (purple line). All models included the random intercept and adjusted for age, sex, race/ethnicity, education levels, poverty income ratio, body mass index, smoking status), alcohol consumption, recreational physical activity, history of diabetes, and chronic kidney disease history.

**Figures 1** and **2** show the overall associations of the four TFAs with SBP and DBP, respectively. The BKMR model indicated that higher plasma concentrations of the TFA mixtures were associated with increased SBP and DBP. Both SBP and DBP increased when all TFAs were fixed at their 30^th^ percentile or above, compared to the 25^th^ percentile of the TFAs mixtures. For example, in comparison to the 25^th^ percentile, the 75^th^ percentile of the TFAs mixtures was associated with a 3.00 (95% CrI: 1.94, 4.06) mm Hg increase in SBP, and a 2.49 (95% CrI: 1.70, 3.29) mm Hg increase in DBP.

Regarding the single effects of the four TFAs on SBP and DBP, the PIP results for each TFA on SBP and DBP are shown in **Supplementary Table S1**. Linolelaidic acid had the highest PIP (PIP_SBP_: 0.96, PIP_DBP_: 1.00), indicating that linolelaidic acid contributed the most to the increased SBP and DBP (**Figures 1**, **2**). An interquartile range (IQR) increase in linolelaidic acid concentration was associated with an increase of 2.15 (95% CrI: 0.69, 3.61), 2.23 (95% CrI: 0.81, 3.65), and 2.31 (95% CrI: 0.87, 3.75) in SBP, and an increase of 2.20 (95% CrI: 1.10, 3.29), 2.24 (95% CrI: 1.18, 3.30), and 2.29 (95% CrI: 1.21, 3.37) in DBP when the other TFA concentrations were simultaneously fixed at their 25^th^, 50^th^, or 75^th^ percentile, respectively. We did not observe significant single effects for the other three TFAs because of their lower PIP values (**Supplementary Table S1**).

We found that the slopes of the dose-response curves of certain TFAs were similar at different percentiles of other TFAs, with others fixed at their middle levels (**Supplementary Figure S4**, **S5**), indicating there were no interactions among the four TFAs.

### 3.5 Associations of TFAs with hypertension in modified Poisson regression models

After adjusting for covariates, higher plasma TFA concentrations were associated with a higher risk of hypertension, although statistically significant associations were only observed for vaccenic and linolelaidic acids with hypertension (vaccenic acid: RR = 1.16, 95% CI: 1.07, 1.25; linolelaidic acid: RR = 1.23, 95% CI: 1.12, 1.36, **Table 4**). Additionally, the highest quartiles of elaidic and linolelaidic acid concentrations were associated with the highest risk (**Table 4**). Adults with plasma concentrations of elaidic acid and linolelaidic acid in the highest quartile had 1.23-fold (95% CI:1.08, 1.39) and 1.34-fold (95% CI:1.18, 1.53) higher risks of hypertension than those in the first quartile.

**Table 4:** Associations of plasma trans fatty acid concentrations with the risk of hypertension in Poisson regression model with robust variance estimates.

Trans-fatty acid	Crude RR (95% CI)	*P*-value	Adjusted RR (95% CI)*	*P*-value
Palmitelaidic acid				
Quartile1	Ref	Ref	Ref	Ref
Quartile2	1.01 (0.88, 1.17)	0.85	0.98 (0.87, 1.11)	0.78
Quartile3	1.17 (1.03, 1.34)	0.02	1.02 (0.90, 1.15)	0.80
Quartile4	1.28 (1.12, 1.45)	<0.01	1.05 (0.93, 1.19)	0.40
Continuous	1.28 (1.15, 1.42)	<0.01	1.07 (0.97, 1.18)	0.19
Vaccenic acid				
Quartile1	Ref	Ref	Ref	Ref
Quartile2	1.05 (0.91, 1.21)	0.51	0.98 (0.87, 1.10)	0.74
Quartile3	1.16 (1.01, 1.33)	0.03	1.06 (0.94, 1.19)	0.36
Quartile4	1.29 (1.13, 1.47)	<0.01	1.10 (0.97, 1.24)	0.12
Continuous	1.17 (1.07, 1.28)	<0.01	1.08 (0.99, 1.17)	0.08
Elaidic acid				
Quartile1	Ref	Ref	Ref	Ref
Quartile2	1.19 (1.03, 1.39)	0.02	1.1 (0.96, 1.25)	0.17
Quartile3	1.35 (1.17, 1.56)	<0.01	1.13 (0.99, 1.28)	0.07
Quartile4	1.65 (1.45, 1.89)	<0.01	1.23 (1.08, 1.39)	<0.01
Continuous	1.39 (1.28, 1.50)	<0.01	1.16 (1.07, 1.25)	<0.01
Linolelaidic acid				
Quartile1	Ref	Ref	Ref	Ref
Quartile2	1.36 (1.18, 1.58)	<0.01	1.27 (1.12, 1.44)	<0.01
Quartile3	1.36 (1.18, 1.58)	<0.01	1.19 (1.04, 1.35)	0.01
Quartile4	1.57 (1.37, 1.81)	<0.01	1.34 (1.18, 1.53)	<0.01
Continuous	1.40 (1.27, 1.54)	<0.01	1.23 (1.12, 1.36)	<0.01

*Adjusting for age, sex, race/ethnicity, education levels, poverty income ratio, body mass index, smoking status), alcohol consumption, recreational physical activity, history of diabetes, and chronic kidney disease history.

### 3.6 Associations of TFAs with hypertension in the BKMR models

**Figure 3:**
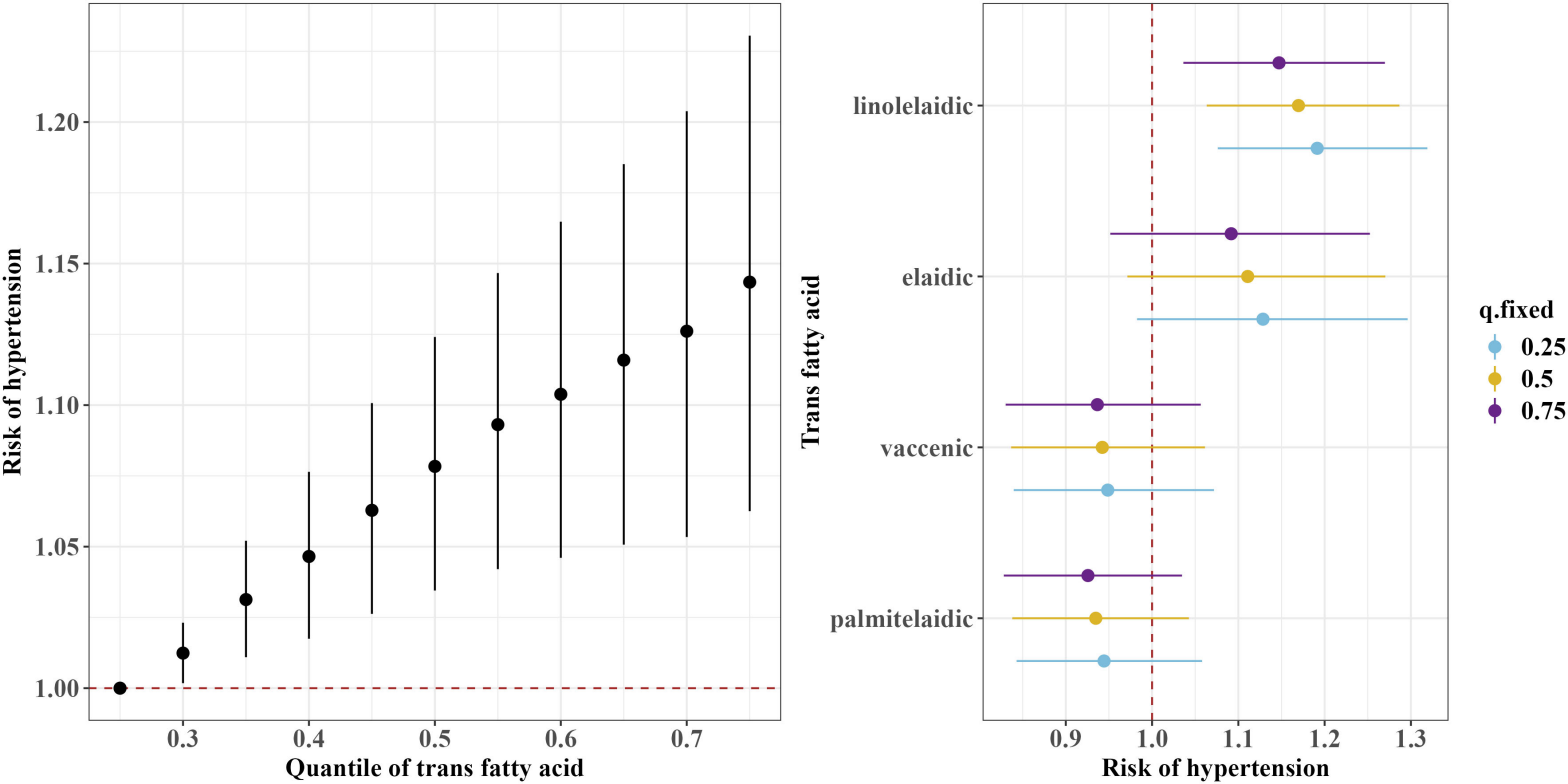
Overall and single-exposure effects of trans fatty acid on the risk of the threshold of hypertension (≥130/80 mm Hg) in Bayesian kernel machine regression models. The left figure shows the effects of four trans fatty acids mixture exposure on the risk of hypertension. The overall associations of TFAs mixture with the risk of hypertension were visualized by the point estimates and their 95% CrI for the difference in the risk of hypertension when all TFAs in the mixture were fixed at the 25^th^ and 75^th^ percentile (per 5 percentile interval), as compared to when they were all fixed at their 25^th^ percentile. The right figure shows the single-exposure effect of TFAs on the risk of hypertension. The single-exposure effect show the specific point estimates and their 95% CrI for the difference in the risk of hypertension for an interquartile range in individual TFA between the 25^th^ and 75^th^ percentile when all the other trans fatty acid were fixed at either the 25^th^ (blue line), 50^th^ (yellow line), or 75^th^ percentile (purple line). All models included the random intercept and adjusted for age, sex, race/ethnicity, education levels, poverty income ratio, body mass index, smoking status), alcohol consumption, recreational physical activity, history of diabetes, and chronic kidney disease history.

The BKMR model showed a significant overall association of the TFAs mixture with an increased risk of hypertension when all TFAs were at or above the 30^th^ percentile compared to their 25^th^ percentile (**Figure 3**). Furthermore, consistent with the results of the continuous blood pressure measurement, the BKMR model also identified linolelaidic acid (PIP=0.93) as the most important contributor to an increased risk of hypertension (**Figure 3**).

### 3.7 Sensitivity analyses

Similar positive associations of the four TFAs with SBP and DBP were also observed when we used a survey-weighted linear regression model (**Supplementary Table S2**). When we repeated the analyses among the population in 1999-2000, the pattern of association between TFAs and blood pressure did not differ by cycles (**Supplementary Figure S6**-**S8**). Moreover, linolelaidic acid also contributed most to the observed associations. Similar results were observed for the analyses when we additionally adjusted for ASCVD history (**Supplementary Tables S3**, **S4**).

## 4 Discussion

In the present study, plasma linolelaidic acid concentrations were associated with higher SBP and DBPas well as an increased risk of hypertension. Additionally, we found that TFA mixture exposure was associated with increased SBP, DBP, and risk of hypertension in adults in the United States, with linolelaidic acid being the most important contributor. These findings were strengthened when similar associations were observed using various analytical strategies.

Higher blood pressure is strongly and independently associated with the risk of CVD, CKD, and all-cause mortality. It is estimated that for every 10-mm Hg increase in SBP, there is a 53% higher risk of atherosclerotic cardiovascular disease among healthy individuals without hypertension or atherosclerotic cardiovascular disease (27). A J-curve relationship between DBP and adverse cardiovascular outcomes has been previously reported (28, 29). Dietary habits, physical activity, and alcohol and smoke consumption have been recognized as major modifiable risk factors of high blood pressure. In our study, we not only found that a one-unit increase in TFA levels was associated with a 2–6 mmHg increase in diastolic and systolic blood pressure, but also showed that TFA levels were associated with an increased risk of clinically defined hypertension. Considering that high levels of TFA can still be found in certain foods (e.g., biscuits) (11), our study provides preliminary evidence that preventing hypertension through dietary fatty acids might be beneficial for public health to promote cardiovascular health.

Most prospective population-based studies have examined the associations between dietary fatty acid intake and the risk of hypertension. Some studies reported that TFA intakes assessed using a food frequency questionnaire were not significantly related to the risk of hypertension (30–32), whereas others reported that TFA intake was associated with an increased risk of hypertension (33). Notably, the use of dietary questionnaires to assess dietary fatty acid intake may be prone to information bias. As the outcome did not occur at the time of exposure assessment, this misclassification is more likely to be non-differential, which may have contributed to the null association between dietary TFA intake from the questionnaire and hypertension risk. Plasma fatty acid is a reasonably accurate and objective marker of dietary fat composition and may reflect an individual’s biological response to a given pattern of dietary fat intake (34, 35). Few studies have examined the association between TFAs biomarkers and the risk of developing hypertension (15, 16). Consistent with the reports by Zhang et al., our study showed that higher plasma TFA concentrations are associated with a higher risk of hypertension (16). One study conducted in Norway, which included individuals born in 1950 with a mean age of 63.9 years older adults, evaluated the sum of different TFAs, and reported that both ruminant and industrial plasma TFAs concentrations were inversely associated with SBP and DBP (15). The inconsistency in the association between TFA exposure and hypertension risk can be attributed to variations in the demographics of the population, different exposure levels, and different sources of TFAs. Additionally, the present study initially reported that exposure to a TFAs mixture was associated with increased blood pressure and the risk of hypertension.

Although the exact mechanism is not completely understood, several potential mechanisms have been proposed. Inflammation is believed to be involved in the initiation and promotion of high blood pressure resulting from TFAs exposure. Randomized clinical trials and observational studies indicated that TFAs are associated with systematic inflammation, characterized by elevated C-reactive protein and pro-inflammatory cytokines such as tumor necrosis factor-αand interleukin 6 (36). Population-based studies have also demonstrated an association between elevated levels of inflammatory markers and the risk of incident hypertension (13). TFAs are also harmful to cells because they modulate endoplasmic reticulum stress, induce reactive oxygen species, and cause oxidative stress (37). *In vitro* evidence suggests that cultured vascular smooth muscle cells and arteries isolated from hypertensive rats and humans show enhanced reactive oxygen species production, amplified redox-dependent signaling, and reduced antioxidant bioactivity (14).

The correlations between palmitelaidic (C16:1 t9), elaidic (C18:1 t9), vaccenic (C18:1 t11), and linolelaidic acids (C18:2 t9, 12) were moderate to high; therefore, we used BKMR models instead of traditional regression models to separate their effects from each other. Our findings provided new evidence that linolelaidic acid exhibits more deleterious effects on hypertension than other TFAs. However, whether different TFAs dominate different effects remains unclear. For instance, some studies have suggested that TFA C18:2 t has greater health hazards in all-cause and CVD-related mortality (10, 38), while others have reported that TFA C18:1 t is the most strongly associated risk factor for CVD death (17). Most previous studies have only evaluated the sum of TFAs according to the types of isomers. Therefore, it is difficult to directly compare those results to ours. *In vitro* studies have suggested that linolelaidic acid induces a stronger lesion effect on apoptosis, cell cycle arrest, and inflammation than elaidic acid (39). Moreover, the position of the trans-double bond could influence how fatty acids are absorbed, perceived, metabolized, and integrated into cellular structures and membranes (8). However, these mechanisms have not been adequately elucidated and require further investigation.

To the best of our knowledge, this is the first study to comprehensively assess the association between blood pressure and single and overall plasma TFA concentrations. Next, the present study evaluated the associations of individual TFAs with hypertension and the association with blood pressure, which may enable researchers to identify more subtle effects of TFAs on cardiovascular health rather than overt clinical disease (40). Additionally, our study used a nationally representative sample of US adults, including a relatively large population, which enhanced the generalizability of our findings. However, there are some limitations should be noted. First, it was difficult to infer a causal association between plasma TFA concentrations and the risk of hypertension owing to the nature of the cross-sectional study design. Further prospective studies are required to confirm these findings. Next, the present study with four TFAs involved a relatively large number of statistical analyses, which increased the type I errors resulting from multiple testing. However, consistent positive associations between TFAs and blood pressure were observed not only in multivariate linear regression models but also in BKMR models, which made them less prone to the issue of multiple comparisons. Finally, the influence of the residual confounding effects of other healthy lifestyles and shared genetic backgrounds cannot be excluded, although we evaluated the effects of numerous confounders.

## 5 Conclusions

Our findings provide preliminary evidence that plasma TFA concentrations are associated with increased blood pressure and the risk of hypertension in US adults. This study also suggests that linolelaidic acid might exhibit more deleterious effects on hypertension than other TFAs. Further studies should be conducted to validate these results.

## Data availability statement

The raw data supporting the conclusions of this article will be made available by the authors, without undue reservation.

## Ethics statement

The studies involving humans were approved by National Center for Health Statistics Research Ethics Review Board. The studies were conducted in accordance with the local legislation and institutional requirements. The participants provided their written informed consent to participate in this study.

## Author contributions

ML: Data curation, Formal analysis, Funding acquisition, Writing – original draft, Writing – review & editing. YT: Conceptualization, Formal analysis, Methodology, Writing – review & editing. DY: Funding acquisition, Writing – review & editing. SL: Writing – review & editing.
