# Supplementary material for: Association of plasma trans fatty acid concentrations with blood pressure and hypertension in U.S. adults
Source: Front Endocrinol (Lausanne). 2024 Apr 22;15:1373095. doi: 10.3389/fendo.2024.1373095 (PMC11070464; doi:10.3389/fendo.2024.1373095)
Supplement: Supplementary file 1 [file DataSheet_1.docx]

**Association of plasma** **trans fatty acid concentrations with blood pressure and hypertension in U.S. adults**

Min Luan^1, *, †^, Youping Tian^2, †^, Dandan Yan^3^, Shuang Liang^4^

^1^Clinical Research Center, Shanghai Sixth People's Hospital Affiliated to Shanghai Jiao Tong University School of Medicine, Shanghai, 200233, China

^2^National Management Office of Neonatal Screening Project for Congenital Heart Disease (CHD), Children's Hospital of Fudan University, National Children's Medical Center, Shanghai, 201102, China

^3^Department of Endocrinology and Metabolism, Shanghai Sixth People's Hospital Affiliated to Shanghai Jiao Tong University School of Medicine, Shanghai Diabetes Institute, Shanghai Clinical Center of Diabetes, Shanghai Key Laboratory of Diabetes Mellitus, Shanghai Key Clinical Center for Metabolic Disease, Shanghai 200233, China

^4^Department of Obstetrics and Gynecology, Shanghai Sixth People's Hospital Affiliated to Shanghai Jiao Tong University School of Medicine, Shanghai, 200233, China

^#^Min Luan and Youping Tian contributed equally to this work and shared the first authorship.

**^*^Correspondence:** Min Luan, Shanghai Sixth People's Hospital Affiliated to Shanghai Jiao Tong University School of Medicine, Shanghai, 200233, China. (min.luan@sjtu.edu.cn)

**Contents of Supplementary Materials**

**Figure S1** Study population of the present study from the NHANES 2009-2010

**Figure S2** Non-linear associations of ln-transformed concentrations of palmitelaidic acid and vaccenic acid with systolic blood pressure in restricted cubic spline models.

**Figure S3** Pearson correlation coefficients between pairs of trans fatty acid concentrations after logarithmic transformation

**Figure S4** Bivariate exposure–response relationship for each trans fatty acid presented on systolic blood pressure when the corresponding trans fatty acid on the right longitudinal axis fixed at 10th, 50th, and 90th percentiles and the remaining trans fatty acid held at 50th percentiles.

**Figure S5** Bivariate exposure–response relationship for each trans fatty acid presented on diastolic blood pressure when the corresponding trans fatty acid on the right longitudinal axis fixed at 10th, 50th, and 90th percentiles and the remaining trans fatty acid held at 50th percentiles

**Figure S6** Overall and single-exposure effects of trans fatty acid on systolic blood pressure among adults in cycle 1999-2000 in Bayesian kernel machine regression models

**Figure S7** Overall and single-exposure effects of trans fatty acid on diastolic blood pressure among adults in cycle 1999-2000 in Bayesian kernel machine regression models

**Figure S8** Overall and single-exposure effects of trans fatty acid on the risk of hypertension among adults in cycle 1999-2000 in Bayesian kernel machine regression models

**Table S1** Posterior inclusion probabilities of Bayesian kernel machine regression models

**Table S2** Associations of plasma trans fatty acid concentrations with systolic blood pressure and diastolic blood pressure with sampling weights in multiple linear regression models

**Table S3** Associations of plasma trans fatty acid concentrations with systolic blood pressure and diastolic blood pressure after additionally adjusting for atherosclerotic cardiovascular disease history in multivariate linear regression models.

**Table S4** Associations of plasma trans fatty acid concentrations with the risk of hypertension after additionally adjusting for atherosclerotic cardiovascular disease history in Poisson regression model with robust variance estimates.


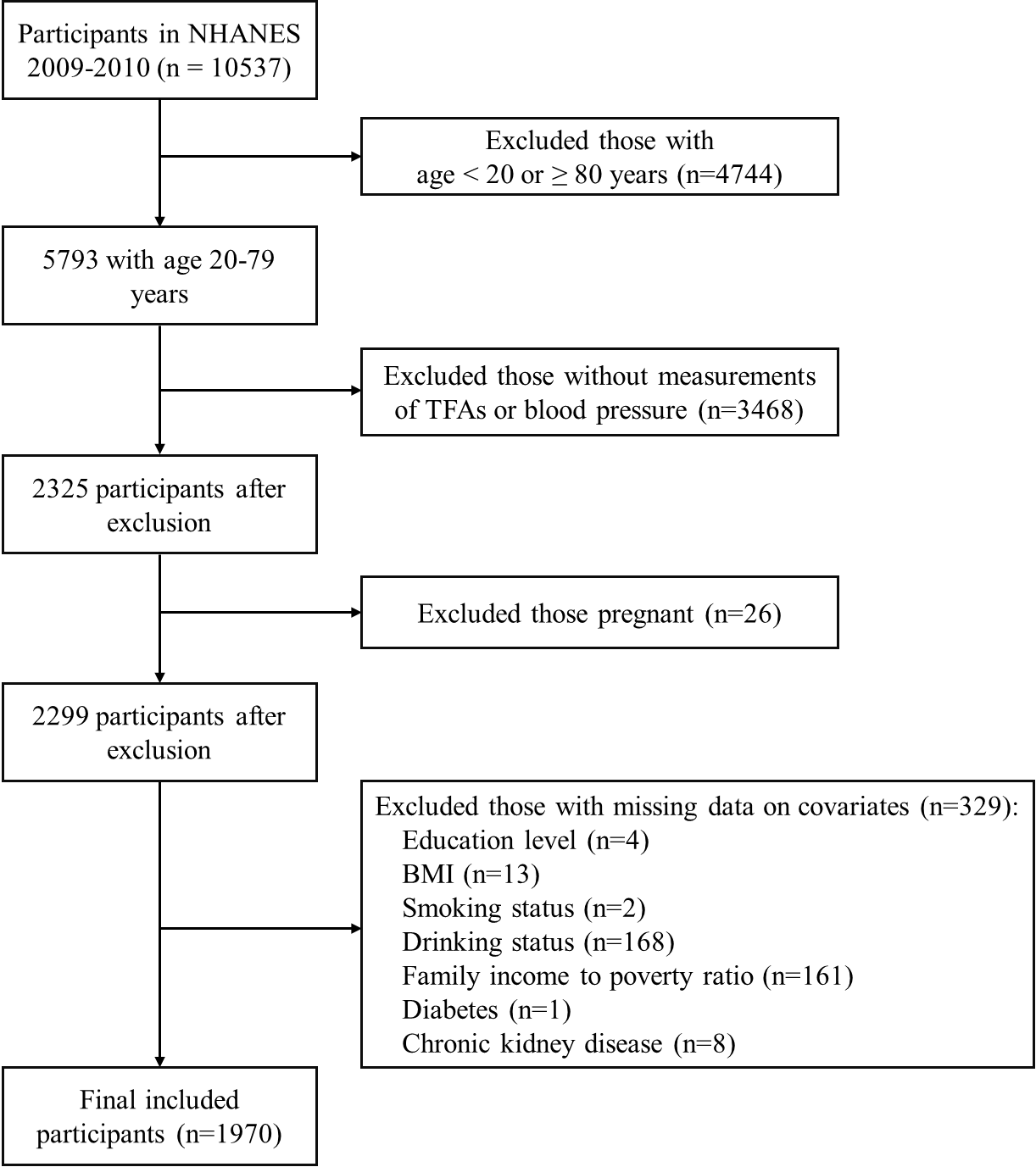


**Figure S1** Study population of the present study from the NHANES 2009-2010


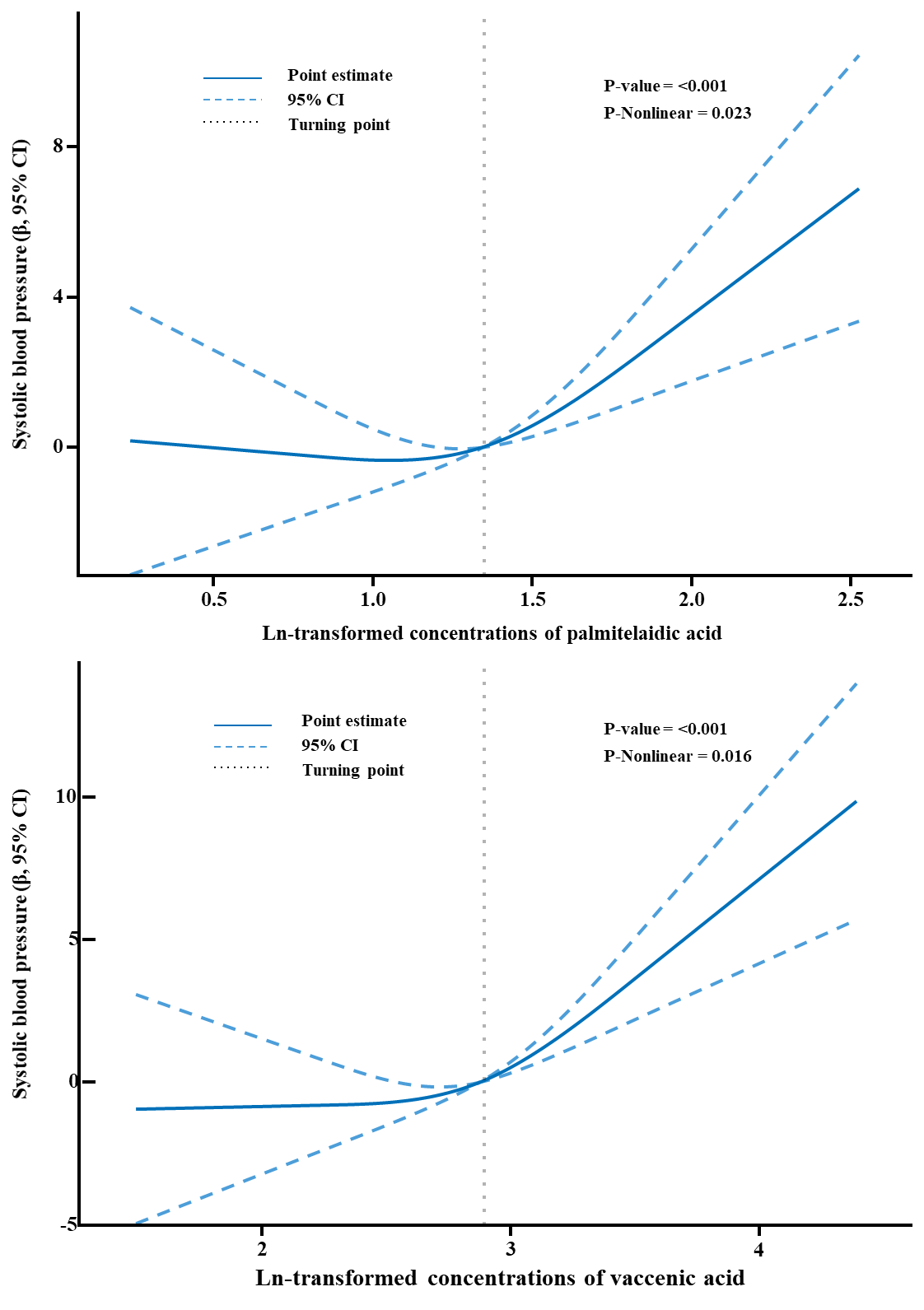


**Figure S2** Non-linear associations of ln-transformed concentrations of palmitelaidic acid and vaccenic acid with systolic blood pressure in restricted cubic spline models.

Adjusting for age (continuous), sex (male and female), race/ethnicity (Mexican American, other Hispanic, Non-Hispanic White, Non-Hispanic Black, and other races), education (less than high school, high school graduate/GED or equivalent, and college or above), poverty income ratio (< 1.30, 1.30–1.85, and ≥1.85), body mass index (< 25 kg/m^2^, 25–30 kg/m^2^, and ≥30 kg/m^2^), smoking status (yes and no), alcohol consumption (yes and no), recreational physical activity (vigorous, moderate, or no activity), diabetes (yes and no), and chronic kidney disease (yes and no).


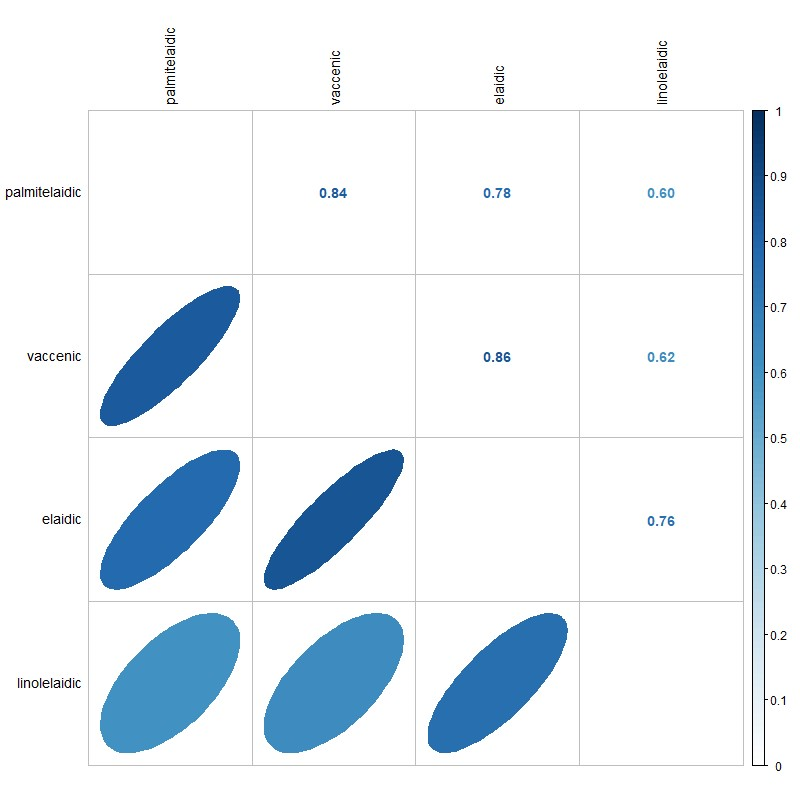


**Figure S3** Pearson correlation coefficients between pairs of trans fatty acid concentrations after logarithmic transformation.


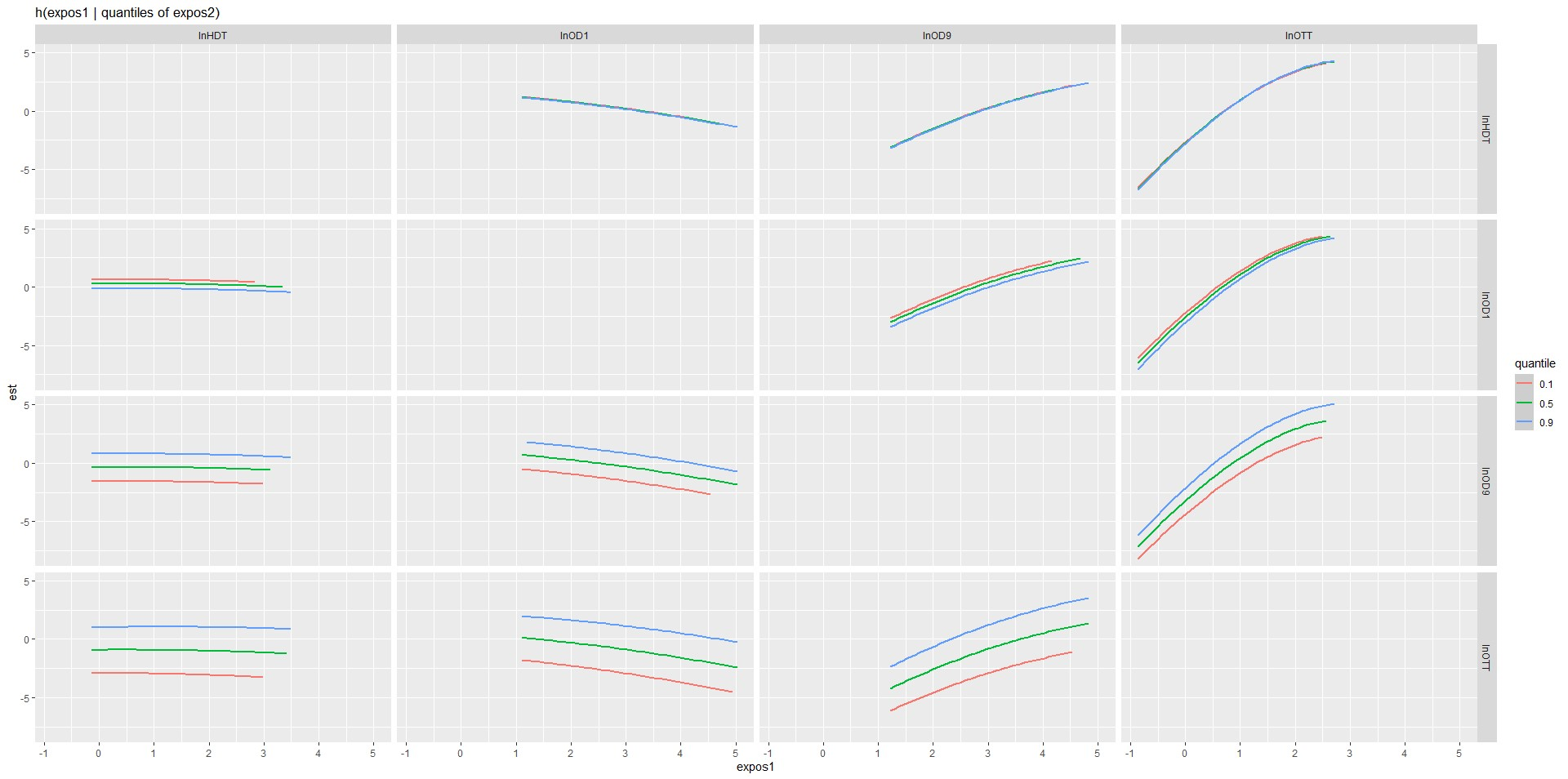


**Figure S4** Bivariate exposure–response relationship for each trans fatty acid presented on systolic blood pressure when the corresponding trans fatty acid on the right longitudinal axis fixed at 10th, 50th, and 90th percentiles and the remaining trans fatty acid held at 50th percentiles.


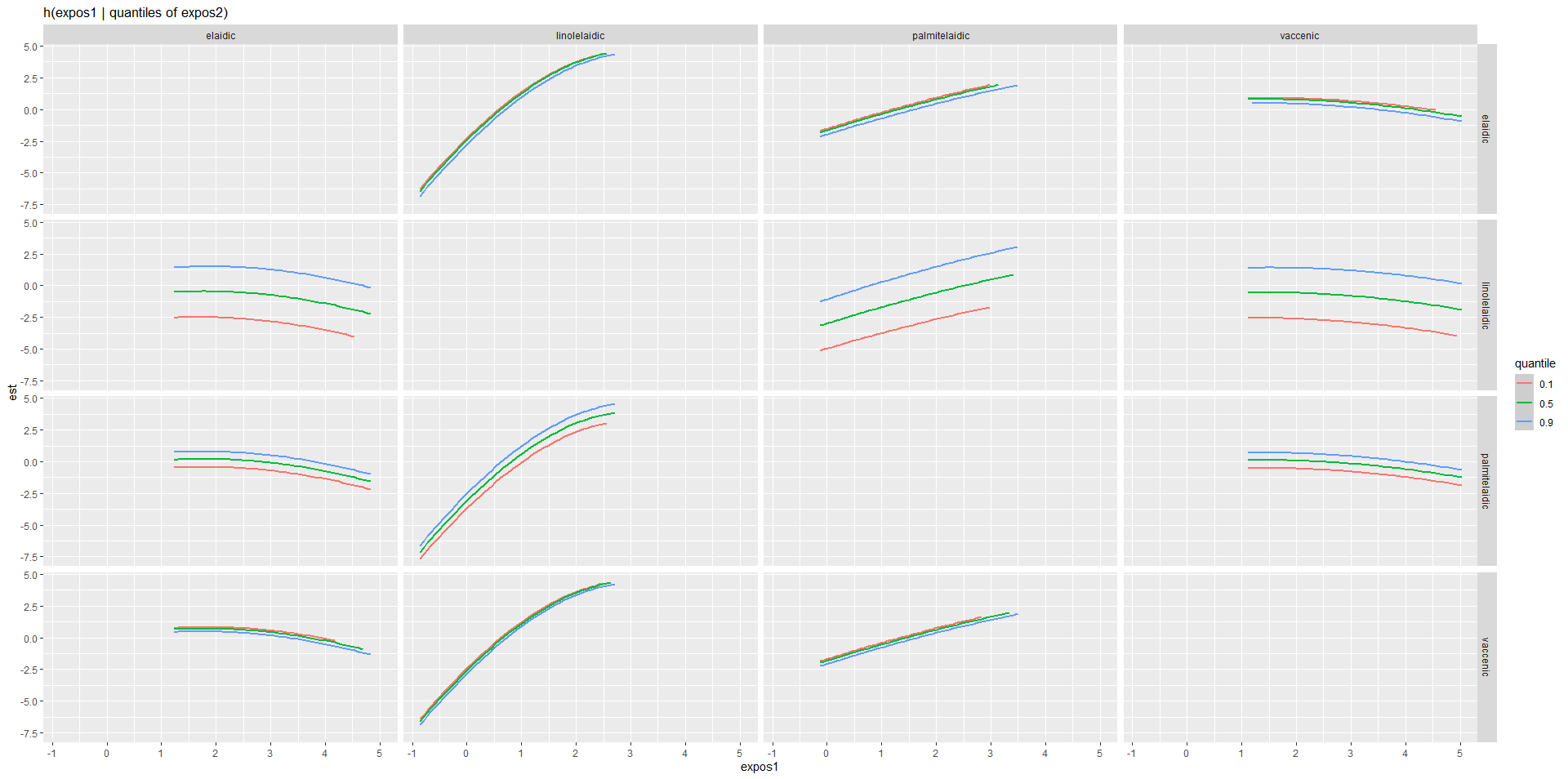


**Figure S5** Bivariate exposure–response relationship for each trans fatty acid presented on diastolic blood pressure when the corresponding trans fatty acid on the right longitudinal axis fixed at 10th, 50th, and 90th percentiles and the remaining trans fatty acid held at 50th percentiles


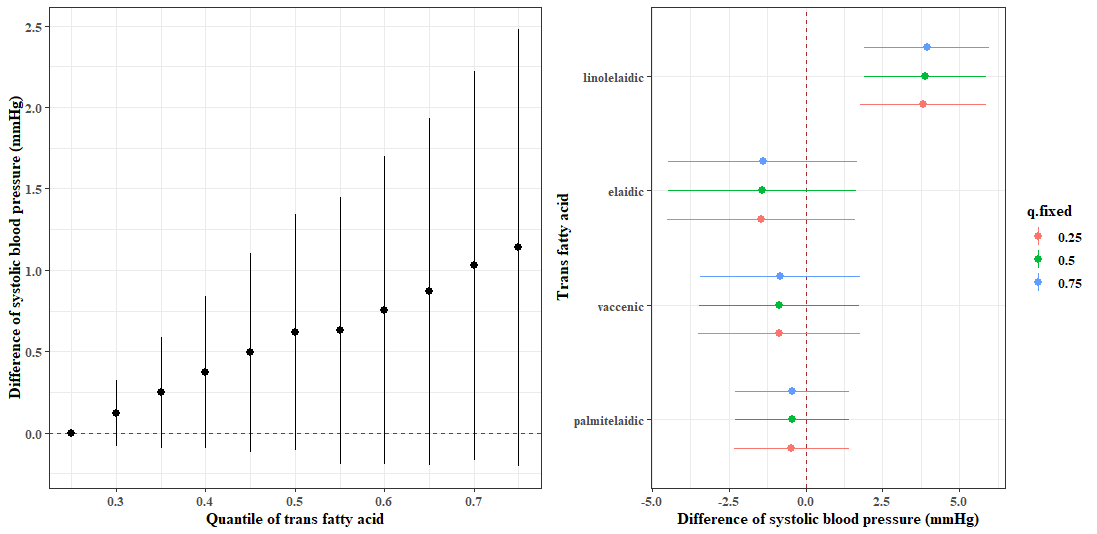


**Figure S6** Overall and single-exposure effects of trans fatty acid on systolic blood pressure among adults in cycle 1999-2000 in Bayesian kernel machine regression models

All models included the random intercept and adjusted for age, sex, race/ethnicity, education levels, poverty income ratio, body mass index, smoking status, alcohol consumption, recreational physical activity, history of diabetes, and chronic kidney disease history.


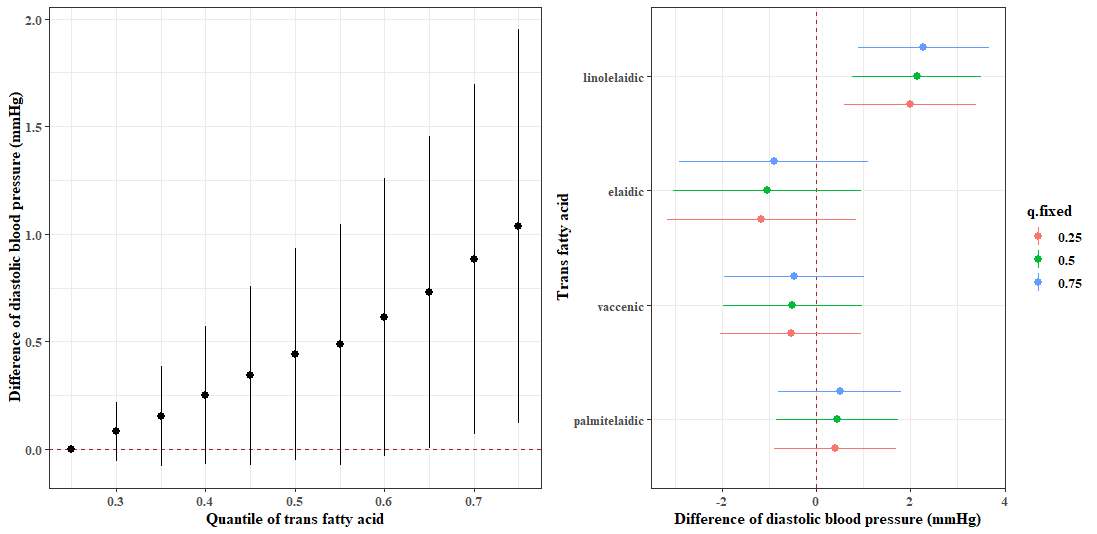


**Figure S7** Overall and single-exposure effects of trans fatty acid on diastolic blood pressure among adults in cycle 1999-2000 in Bayesian kernel machine regression models

All models included the random intercept and adjusted for age, sex, race/ethnicity, education levels, poverty income ratio, body mass index, smoking status, alcohol consumption, recreational physical activity, history of diabetes, and chronic kidney disease history.


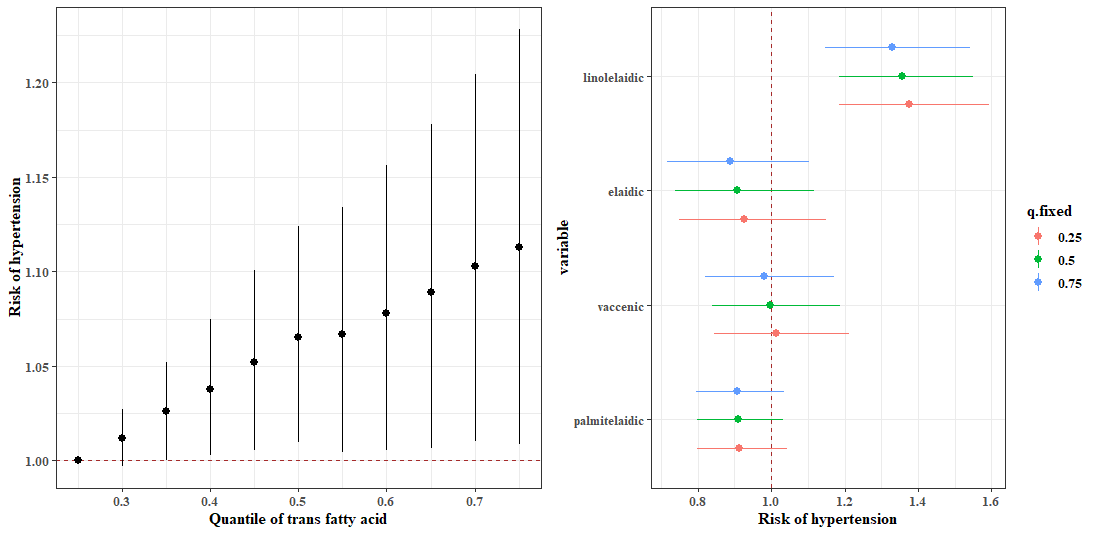


**Figure S8** Overall and single-exposure effects of trans fatty acid on the risk of hypertension among adults in cycle 1999-2000 in Bayesian kernel machine regression models

All models included the random intercept and adjusted for age, sex, race/ethnicity, education levels, poverty income ratio, body mass index, smoking status, alcohol consumption, recreational physical activity, history of diabetes, and chronic kidney disease history.

**Table S1** Posterior inclusion probabilities of Bayesian kernel machine regression models

|  | PIP | | | |
| --- | --- | --- | --- | --- |
|  | Palmitelaidic acid | Vaccenic acid | Elaidic acid | Linolelaidic acid |
| Systolic blood pressure | 0.02 | 0.03 | 0.09 | 0.96 |
| Diastolic blood pressure | 0.05 | 0.07 | 0.11 | 1.00 |
| Hypertension | 0.45 | 0.35 | 0.40 | 0.93 |

**Table S2** Associations of plasma trans fatty acid concentrations with systolic blood pressure and diastolic blood pressure with sampling weights in multiple linear regression models

| Trans fatty acid | Systolic blood pressure | |  | Diastolic blood pressure | |
| --- | --- | --- | --- | --- | --- |
|  | β (95% CI) | P-value |  | β (95% CI) | P-value |
| Palmitelaidic acid |  |  |  |  |  |
| Quartile1 | Ref | Ref |  | Ref | Ref |
| Quartile2 | 0.13 (-1.77, 2.03) | 0.89 |  | 0.88 (-0.57, 2.33) | 0.23 |
| Quartile3 | 1.98 (-0.41, 4.36) | 0.11 |  | 1.96 (0.58, 3.33) | 0.01 |
| Quartile4 | 2.05 (-0.69, 4.78) | 0.15 |  | 3.66 (1.81, 5.51) | <0.01 |
| Continuous | 2.68 (0.67, 4.69) | 0.02 |  | 3.17 (1.63, 4.70) | <0.01 |
| Vaccenic acid |  |  |  |  |  |
| Quartile1 | Ref | Ref |  | Ref | Ref |
| Quartile2 | 0.05 (-2.05, 2.15) | 0.96 |  | 1.59 (-0.31, 3.50) | 0.11 |
| Quartile3 | 1.07 (-0.40, 2.54) | 0.16 |  | 2.72 (1.54, 3.91) | <0.01 |
| Quartile4 | 2.58 (0.80, 4.37) | 0.01 |  | 3.25 (1.92, 4.58) | <0.01 |
| Continuous | 2.28 (0.75, 3.81) | <0.01 |  | 2.13 (0.90, 3.36) | <0.01 |
| Elaidic acid |  |  |  |  |  |
| Quartile1 | Ref | Ref |  | Ref | Ref |
| Quartile2 | 2.84 (0.9, 4.77) | <0.01 |  | 1.79 (0.43, 3.14) | 0.02 |
| Quartile3 | 1.45 (-0.60, 3.49) | 0.17 |  | 2.16 (0.44, 3.89) | 0.02 |
| Quartile4 | 4.11 (2.41, 5.80) | <0.01 |  | 3.25 (1.44, 5.05) | <0.01 |
| Continuous | 3.19 (1.71, 4.68) | <0.01 |  | 2.41 (1.00, 3.82) | <0.01 |
| Linolelaidic acid |  |  |  |  |  |
| Quartile1 | Ref | Ref |  | Ref | Ref |
| Quartile2 | 2.45 (0.68, 4.23) | 0.01 |  | 2.66 (1.50, 3.83) | <0.01 |
| Quartile3 | 2.46 (-0.59, 5.52) | 0.12 |  | 2.73 (0.78, 4.69) | 0.01 |
| Quartile4 | 5.00 (2.38, 7.63) | <0.01 |  | 4.75 (2.19, 7.30) | <0.01 |
| Continuous | 4.29 (1.77, 6.82) | <0.01 |  | 3.85 (1.82, 5.89) | <0.01 |

Adjusting for age (continuous), sex (male and female), race/ethnicity (Mexican American, other Hispanic, Non-Hispanic White, Non-Hispanic Black, and other races), education (less than high school, high school graduate/GED or equivalent, and college or above), poverty income ratio (< 1.30, 1.30–1.85, and ≥1.85), body mass index (< 25 kg/m^2^, 25–30 kg/m^2^, and ≥30 kg/m^2^), smoking status (yes and no), alcohol consumption (yes and no), recreational physical activity (vigorous, moderate, or no activity), diabetes (yes and no), and chronic kidney disease (yes and no).

**Table S3** Associations of plasma trans fatty acid concentrations with systolic blood pressure and diastolic blood pressure after additionally adjusting for atherosclerotic cardiovascular disease history in multivariate linear regression models.

| **Trans fatty acid** | **Systolic blood pressure** | |  | **Diastolic blood pressure** | |
| --- | --- | --- | --- | --- | --- |
|  | **β (95% CI)*** | ***P*-value** |  | **β (95% CI)*** | ***P*-value** |
| Palmitelaidic acid |  |  |  |  |  |
| Quartile1 | Ref | Ref |  | Ref | Ref |
| Quartile2 | -1.06 (-2.98, 0.86) | 0.28 |  | -0.05 (-1.49, 1.38) | 0.94 |
| Quartile3 | 0.49 (-1.47, 2.45) | 0.62 |  | 1.57 (0.11, 3.03) | 0.04 |
| Quartile4 | 2.32 (0.32, 4.32) | 0.02 |  | 2.66 (1.17, 4.15) | <0.01 |
| Continuous | 2.88 (1.21, 4.56) | <0.01 |  | 2.78 (1.54, 4.02) | <0.01 |
| Vaccenic acid |  |  |  |  |  |
| Quartile1 | Ref | Ref |  | Ref | Ref |
| Quartile2 | -0.20 (-2.13, 1.72) | 0.84 |  | 1.20 (-0.23, 2.64) | 0.10 |
| Quartile3 | 0.54 (-1.41, 2.48) | 0.59 |  | 2.24 (0.79, 3.69) | <0.01 |
| Quartile4 | 2.96 (0.97, 4.95) | <0.01 |  | 2.60 (1.12, 4.08) | <0.01 |
| Continuous | 2.41 (1.01, 3.80) | <0.01 |  | 2.04 (1.01, 3.08) | <0.01 |
| Elaidic acid |  |  |  |  |  |
| Quartile1 | Ref | Ref |  | Ref | Ref |
| Quartile2 | 2.47 (0.52, 4.42) | 0.01 |  | 1.08 (-0.36, 2.53) | 0.14 |
| Quartile3 | 1.10 (-0.90, 3.09) | 0.28 |  | 2.00 (0.53, 3.48) | 0.01 |
| Quartile4 | 4.78 (2.70, 6.86) | <0.01 |  | 3.47 (1.93, 5.02) | <0.01 |
| Continuous | 3.59 (2.19, 4.99) | <0.01 |  | 2.41 (1.38, 3.45) | <0.01 |
| Linolelaidic acid |  |  |  |  |  |
| Quartile1 | Ref | Ref |  | Ref | Ref |
| Quartile2 | 2.60 (0.62, 4.58) | 0.01 |  | 2.55 (1.08, 4.02) | <0.01 |
| Quartile3 | 2.24 (0.22, 4.26) | 0.03 |  | 2.11 (0.61, 3.62) | 0.01 |
| Quartile4 | 6.03 (3.94, 8.11) | <0.01 |  | 4.48 (2.93, 6.03) | <0.01 |
| Continuous | 4.80 (3.11, 6.49) | <0.01 |  | 3.76 (2.51, 5.01) | <0.01 |

*Adjusting for age, sex, race/ethnicity, education, poverty income ratio, body mass index, smoking status, alcohol consumption, recreational physical activity, history of diabetes, chronic kidney disease history. and atherosclerotic cardiovascular disease.

**Table S4** Associations of plasma trans fatty acid concentrations with the risk of hypertension after additionally adjusting for atherosclerotic cardiovascular disease history in Poisson regression model with robust variance estimates.

| **Trans-fatty acid** | **Adjusted RR (95% CI)*** | ***P*-value** |
| --- | --- | --- |
| Palmitelaidic acid |  |  |
| Quartile1 | Ref | Ref |
| Quartile2 | 0.98 (0.87, 1.11) | 0.78 |
| Quartile3 | 1.02 (0.90, 1.15) | 0.74 |
| Quartile4 | 1.05 (0.93, 1.18) | 0.42 |
| Continuous | 1.07 (0.97, 1.18) | 0.19 |
| Vaccenic acid |  |  |
| Quartile1 | Ref | Ref |
| Quartile2 | 0.98 (0.87, 1.11) | 0.78 |
| Quartile3 | 1.06 (0.94, 1.20) | 0.33 |
| Quartile4 | 1.10 (0.97, 1.24) | 0.13 |
| Continuous | 1.08 (0.99, 1.17) | 0.08 |
| Elaidic acid |  |  |
| Quartile1 | Ref | Ref |
| Quartile2 | 1.10 (0.96, 1.25) | 0.16 |
| Quartile3 | 1.13 (0.99, 1.29) | 0.07 |
| Quartile4 | 1.23 (1.08, 1.40) | <0.01 |
| Continuous | 1.16 (1.07, 1.25) | <0.01 |
| Linolelaidic acid |  |  |
| Quartile1 | Ref | Ref |
| Quartile2 | 1.28 (1.13, 1.45) | <0.01 |
| Quartile3 | 1.20 (1.05, 1.37) | 0.01 |
| Quartile4 | 1.35 (1.19, 1.54) | <0.01 |
| Continuous | 1.24 (1.13, 1.37) | <0.01 |

*Adjusting for age, sex, race/ethnicity, education, poverty income ratio, body mass index, smoking status, alcohol consumption, recreational physical activity, history of diabetes, chronic kidney disease history. and atherosclerotic cardiovascular disease.
